# Body Morphology, Energy Stores, and Muscle Enzyme Activity Explain Cricket Acoustic Mate Attraction Signaling Variation

**DOI:** 10.1371/journal.pone.0090409

**Published:** 2014-03-07

**Authors:** Ian R. Thomson, Charles-A. Darveau, Susan M. Bertram

**Affiliations:** 1 Department of Biology, Carleton University, Ottawa, Ontario, Canada; 2 Department of Biology, University of Ottawa, Ontario, Canada; National Cancer Institute, United States of America

## Abstract

High mating success in animals is often dependent on males signalling attractively with high effort. Since males should be selected to maximize their reproductive success, female preferences for these traits should result in minimal signal variation persisting in the population. However, extensive signal variation persists. The genic capture hypothesis proposes genetic variation persists because fitness-conferring traits depend on an individual's basic processes, including underlying physiological, morphological, and biochemical traits, which are themselves genetically variable. To explore the traits underlying signal variation, we quantified among-male differences in signalling, morphology, energy stores, and the activities of key enzymes associated with signalling muscle metabolism in two species of crickets, *Gryllus assimilis* (chirper: <20 pulses/chirp) and *G. texensis* (triller: >20 pulses/chirp). Chirping *G. assimilis* primarily fuelled signalling with carbohydrate metabolism: smaller individuals and individuals with increased thoracic glycogen stores signalled for mates with greater effort; individuals with greater glycogen phosphorylase activity produced more attractive mating signals. Conversely, the more energetic trilling *G. texensis* fuelled signalling with both lipid and carbohydrate metabolism: individuals with increased β-hydroxyacyl-CoA dehydrogenase activity and increased thoracic free carbohydrate content signalled for mates with greater effort; individuals with higher thoracic and abdominal carbohydrate content and higher abdominal lipid stores produced more attractive signals. Our findings suggest variation in male reproductive success may be driven by hidden physiological trade-offs that affect the ability to uptake, retain, and use essential nutrients, although the results remain correlational in nature. Our findings indicate that a physiological perspective may help us to understand some of the causes of variation in behaviour.

## Introduction

The lek paradox refers to the puzzle of how male sexual traits continually exhibit extensive genetic variation even though female preferences should cause its rapid decline [1]–[4]. Genic capture was introduced as a solution to this paradox. Genic capture posits that preferred traits are highly genetically variable because they depend on many underlying physiological, morphological, and biochemical traits that affect condition [3]. Since most traits are influenced by multiple loci, high genetic variation should be maintained via mutation-selection balance [3], [4].

Genic capture rests on the assumption that genetic variation in condition drives variation in preferred traits. But, what is condition and how should it be measured? Historically, most behavioural ecologists have measured condition by quantifying energy stores, often using a measure of residual mass (mass corrected for body size). Several researchers have expressed concern that the limitations of residual mass make it an inappropriate measure of condition e.g. [4]–[8]. In a recent perspective article, Hill [9] defined condition as “*the relative capacity to maintain optimal functionality of essential cellular processes*”. He argued that condition is influenced by genotype, epigenetic state, and somatic state. Even though somatic state incorporates energy stores, it includes all variables affecting current body state such as age, external influences such as parasite load, toxic load, physical and cellular damage, gut contents, social status, and quality of territory. Hill's [9] definition forces us to move beyond thinking of condition as being reflected in energy stores alone and toward thinking of condition in terms of physiological, cellular, and biochemical processes. Hill [9] called upon behavioural ecologists to ascertain whether expression of preferred traits reflects a capacity to remain near an optimal state. To do this, we must determine which cellular processes link preferred trait production to vital system functionality [9]. Here we explore the cellular processes linked to preferred trait production using field crickets (Orthoptera: Gryllidae) as model organisms.

Male field crickets produce easily quantifiable but highly variable acoustic mate attraction signals [10]–[12]. Signalling is highly heritable in many cricket species [13], [14], including *G. texensis* [15], [16]. Males signal acoustically by raising their forewings and rubbing the scraper (plectrum) on the top of one wing against the file on the underside of the other wing [10], [11]. Sound produced by this stridulatory action is amplified by the harp area of the wing [11], [17], [18]. Each wing's closing stroke produces one pulse of sound, and males concatenate pulses into groups. Chirpers concatenate pulses into short groups (<20 pulses) while trillers concatenate pulses into long groups (>20 pulses; [17], [19], [20]).

Male crickets often exhibit substantial intraspecific variation in their signalling effort (quantity) and in the fine scale structure (quality) of their mating signals [12], [21]–[24]. Female crickets tend to preferentially mate with males that signal with high effort ([25]; *G. integer*: [12]; *G. firmus*: [26]; *G. campestris*: [27]; *Teleogryllus commodus*: [28]; *G. pennsylvanicus*: [29]), at high chirp rates (*G. lineaticeps*: [30]), with long chirp durations (*G. lineaticeps*: [31]) and more or average pulses per chirp/trill (more: *G*. *bimaculatus*: [32]; *G. texensis*: [33]; average: *G. campestris*: [32]). Female preferences should result in directional or stabilizing selection influencing male acoustic signalling traits. This selection should cause a reduction in the variation in male signals, yet substantial variation persists for both signalling effort and fine-scale components. To understand how variation in preferred signalling traits is maintained, we quantified the underlying physiological, morphological, and biochemical processes linking preferred trait production to system functionality [9].

Cricket acoustic mate attraction signalling appears to be powered by aerobic metabolism [34]. Aerobic metabolism in insect muscles has been studied most in the context of flight. Generally, carbohydrates are used as fuel in species that perform high-intensity but short duration flight activity, while prolonged fliers use a combination of carbohydrates and lipids to fuel their flights [35]–[37]. Similar to flight activity, cricket acoustic signalling activity occurs over both short and long time periods (e.g., signalling for 10 minutes versus 16 hours a day) and at varying levels of intensity, depending on whether the male is a chirper or a triller and the number of times he chirps each minute.

Our previous research on European house crickets (*Acheta domesticus*) suggested chirping males use carbohydrates to fuel signalling. We characterized muscle metabolic phenotypes by measuring activities of energy metabolism enzymes and showed that males that signalled with highest effort had highest pyruvate kinase activity, an indicator of glycolytic flux capacity [38]. However, European house crickets incorporate few (2–3) pulses into each chirp and typically signal less often than most other field crickets species [24]. Given *A. domesticus*' low energetic signalling may not be representative of other cricket species, we quantified the cellular processes linking production of acoustic mating signals in two different field cricket species, on chirping male Jamaican field crickets (*Gryllus assimilis*) and on trilling male Texas field crickets (*Gryllus texensis*).

**Figure 1:**
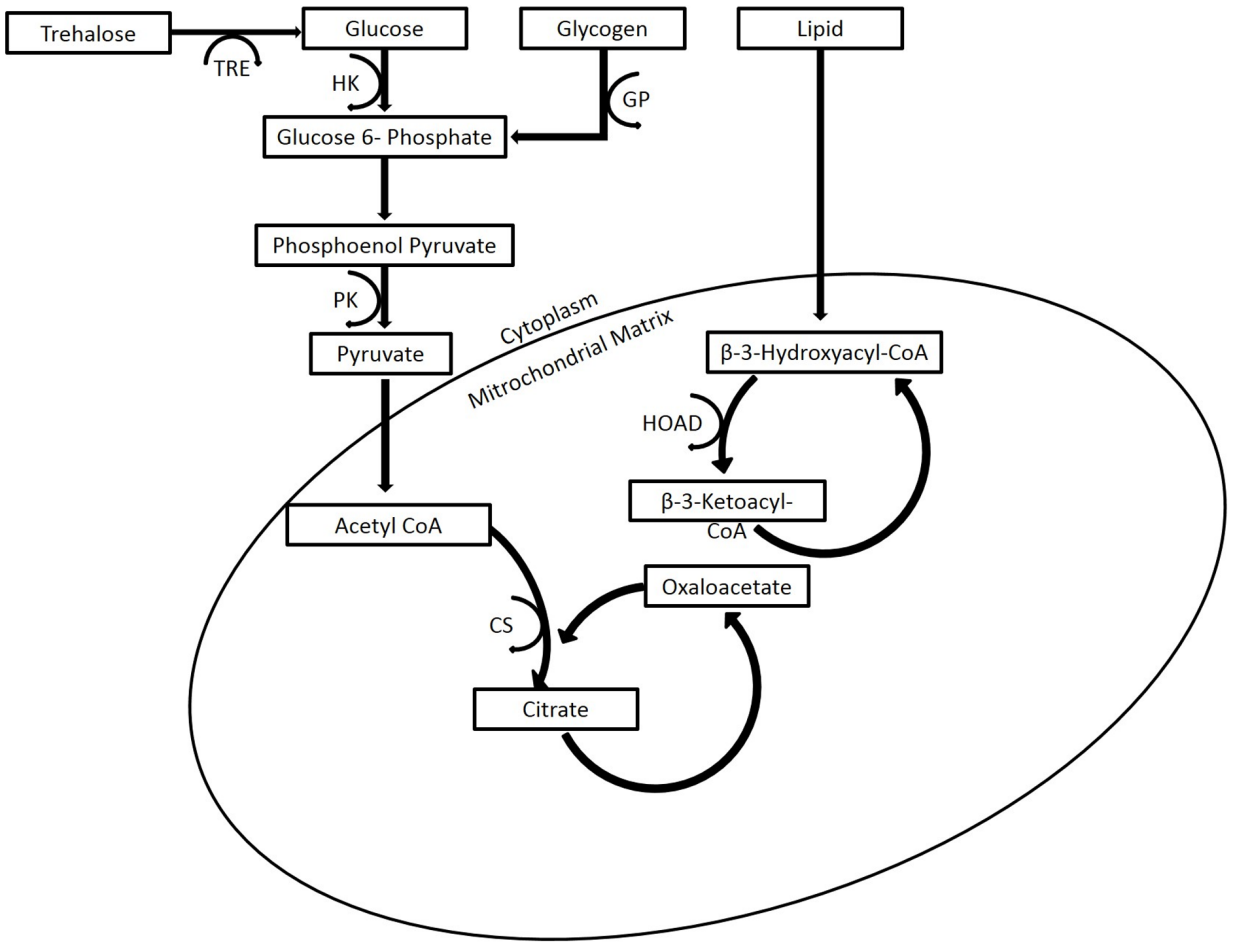
Metabolic pathways of six enzymes used to study muscle metabolism. Abbreviations are as follows: CV (coefficient of variation), PK (pyruvate kinase), GP (glycogen phosphoylase), CS (citrate synthase), HOAD (β-hydroxyacyl-CoA dehydrogenase), and TRE (trahalase)

We hypothesized that 1) intraspecific variation in sexual signalling quantity and quality would be correlated with variation in signalling muscle enzymes associated with energy metabolism, and 2) intraspecific variation in sexual signalling quantity and quality would be correlated with variation in capacity to accumulate and store carbohydrates and/or lipids. To test our hypotheses we quantified intraspecific variation in cricket signalling behaviour over a one-week period and then quantified variation in morphological features and biochemical properties (signalling muscle enzymes activity and fuels stored in the abdomen and thorax). Specifically, we measured variation in maximal activity (*V*_max_) of six enzymes used as indicators of metabolic capacities (Figure 1). To quantify variation in the capacity for carbohydrate use we measured two glycolytic enzymes, hexokinase and pyruvate kinase (HK and PK), as well as the enzymes trehalase and glycogen phosphorylase (TRE and GP), which breakdown trehalose and glycogen into substrates for glycolysis. To assess variation in the capacity for lipid use we measured a commonly used marker for β-oxidation, β-hydroxyacyl-CoA dehydrogenase (HOAD). We also quantified the variation in the mitochondrial enzyme citrate synthase (CS) to measure oxidative phosphorylation capacity. To assess variation in fuel stores we extracted and measured the proportion of free carbohydrate, glycogen, and lipid fractions in the thorax and abdomen.

## Methods

### Ethics Statement

We did not require specific permits for collecting invertebrates because these cricket species are neither endangered nor protected. We thank Steven Gibson and the Stengl Lost Pines Biological Station (latitude ∼30° 17′ N, longitude ∼97° 46′ W, elevation ∼145 m) at the University of Texas for hosting our laboratory during the cricket-collecting trip that resulted in the establishment of our *G. assimilis* and *G. texensis* laboratory populations. Crickets were collected on private land; researchers interested in collecting crickets at Stengl Lost Pines should contact Seven Gibson.

We received a permit from the Canadian Food Inspection Agency (permit # 2007-03130) to import collected crickets into Canada. The crickets were contained in a greenhouse at Carleton University, which is a Canadian Food Inspection Agency certified holding facility with certification level Plant Pest Containment Level 1 (permit # P-2012-03836). While we did not require specific permits to conduct our experiments, our study was conducted in accordance with the guidelines of the Canadian Council on Animal Care.

### Cricket Rearing

*Gryllus assimilis* and *G. texensis* were collected in Bastrop County, Texas, United States, from 15 to 24-September, 2008 and brought back to the laboratories at Carleton University, Ottawa, Canada. The crickets were kept on a light:dark cycle of 14:10 h at 26°C ± 2 °C and fed *ad libitum* water and food (Harland's Teklad Rodent diet 8604; 24.3% protein, 40.2% carbohydrate, 4.7% lipid, 16.4% fiber, 7.4% ash). Crickets were reared from egg to final juvenile instar in communal plastic containers (64 cm x 40 cm x 41.9 cm) and were checked daily for any individuals that had undergone final (imaginal) moult. In 2011 new imaginal moult male *G. assimilis* (*N* = 92) and *G. texensis* (*N = *63) were removed from the colony and housed individually in 500 mL clear plastic containers. The individual containers had lids with a 4 cm x 4 cm section covered with metal screening to allow air and sound to pass through. Each cricket had unbleached crumpled paper towel for shelter and *ad libitum* food and water in his container. Light cycles, temperatures, and diet during the experiment were identical to development.

### Acoustic Recording

Males were transferred into the electronic acoustic recording system (EARS-II; designed and built for our laboratory by Cambridge Electronic Design Ltd., Cambridge, UK) to have their acoustic mate attraction signalling behaviour recorded for a one week period starting at 7 days post imaginal moult (for details, refer to [39]). The EARS-II housed 96 males, each in acoustic foam lined Styrofoam containers that minimized sound contamination from neighbouring crickets. Each container was equipped with a microphone and light (14:10 h light:dark cycle). Acoustic signalling behaviour was monitored continually by CricketSong software (Cambridge Electronic Design Ltd., Cambridge, UK) that dynamically adjusted its amplitude threshold to ensure all sound pulses were recorded. The CricketSong software automatically analyzed the sound wave recorded by the microphone and calculated, in real time, the mean pulse duration (ms), mean interpulse duration (time between pulses; ms), mean number of pulses per chirp, mean chirp duration (ms), mean interchirp duration (time between chirps; ms), mean amplitude (Pa), and mean carrier frequency of the call (Hz). Signal amplitude was converted to decibels (dB) using the equation 20 x Log_10_ (Mean Amp (Pa)/0.00002). CricketSong also measured call output, enabling us to quantify the mean number of pulses produced throughout the day and the mean time spent calling per day from days 7-14 of adulthood. We calculated mean daily number of pulses and time spent calling by averaging these values across all seven days. We used mean signalling values to explore the relationships with morphology and enzyme activity because signalling parameters are highly repeatable within individuals of both species [24].

### Morphometric Analysis and Dissection

Both cricket species were removed from the EARS-II on day 14 post imaginal moult and weighed to the nearest mg using a Denver Instrument PI-114 balance. Morphological characteristics (pronotum width, pronotum length, pronotum trace outline, and head capsule width) were quantified to the nearest µm using a Zeiss Discovery V12 microscope and Axiovision software version 4.8.2.0 (Carl Zeiss MicroImaging, Jena, Germany). We euthanized each cricket by placing it on ice and removing its head with sharp scissors. The head and abdomen were immediately frozen in liquid nitrogen. Each cricket's thorax was dissected ventrally and the mesothoracic signalling muscles (dorsoventral, basalar and subalar) were removed, weighed, and frozen in liquid nitrogen. The rest of the thorax (including metathoracic flight muscles) was frozen in liquid nitrogen. All tissue was stored at −80°C and then transported in dry ice to the University of Ottawa, Ottawa, Canada for biochemical analyses. Wing harp area and file length were quantified using the Zeiss Discovery V12 microscope and Axiovision software version 4.8.2.0. Harp area was measured by tracing the veins that surround the harp [18]. All visible teeth were included when measuring stridulatory file length.

### Enzyme Activity Measurements

Both species' signalling muscles were analysed to determine the activity of energy metabolism enzymes involved in signalling behaviour. Muscle tissues were homogenized according to protocols previously described by Bertram et al. [38]. Maximum activities (*V*_max_) of six different enzymes (GP, PK, CS, TRE, HK and HOAD) were assayed in triplicate at 25°C using a Biotek Synergy 2 plate spectrophotometer (Biotek, Winooski, VT, U.S.A.). The activity of GP, PK, HOAD and CS were measured following conditions described by Bertram et al. [38]. For HK and TRE, assay conditions and substrate concentrations required to elicit *V_max_* were as follows: HK: 100 mM imidazole (pH 8.1), 10 mM of MgCl_2_, 100 mM KCl, 1 mM NADP, 5 mM ATP, 5 mM D-glucose (omitted from control) and 1.25 U of glucose 6-phosphate dehydrogenase; TRE: 100 mM potassium phosphate (pH 6.6), 1.1 mM of MgCl_2_, 0.75 mM of NADP, 1.1 mM ATP, 10 mM trehalose (omitted from control), 1.25 U of glucose 6-phosphate dehydrogenase. 1.25 U of hexokinase. Reactions were monitored using nicotinamide adenine dinucleotide phosphate (NADPH) at 340 nm using a millimolar extinction coefficient of 6.22.

### Quantifying Thoracic and Abdominal Energy Stores

Thirty crickets of each species were selected for body composition analysis. Selected crickets were comprised of equal numbers of high, intermediate, and low effort signallers. Signaller categories were determined by rank ordering all crickets based on their time spent calling. The top 25% from each species were deemed to be high effort signallers, the bottom 25% from each species were deemed to be low effort signallers, while the intermediate 50% (25%–75%) from each species were deemed to be intermediate effort signallers. For each species, ten individuals from each category were selected at random from the group of individuals with complete enzyme assay data.

The methodology for the extraction of lipids, glycogen, and free carbohydrate from cricket samples followed Lorenz [40]. Cricket thoraxes and abdomens were weighed (fresh weight) and homogenized by first mincing with a pair of fine scissors, and then using an Omni-Prep homogenizer with a 7 mm Rotor Stator tip (Omni International, Marietta, GA). All centrifugation was performed for 10 min at 21000 g at 4°C (Sorvall Legend, Thermo Scientific, Waltham, MA).

We used colourimetric determination to measure body energy stores. We performed all colourimetric assays in plastic 96 well plates (Costar 21, Corning, Tewksbury, MA) in triplicate using a Biotek Synergy 2 plate spectrophotometer (Biotek, Winooski, VT). Total lipid was extracted using chloroform and was measured using the phospho-vanillin method [41], with the exception that soybean oil was dissolved in hexane instead of chloroform so that plastic 96-well plates could be used (this modification is unlikely to have affected the results because the extracted lipid was also ultimately dissolved in hexane as per Lorenz [40]). Individual standard curves were made for each plate that was read. Total glycogen and free carbohydrate were measured using the anthrone method using anhydrous glucose as the standard [41]. All values obtained from the spectrophotometer were compared to standard curves and total lipid, glycogen, and carbohydrate amounts were calculated for both the thorax and abdomen of each cricket. These values were then converted and presented as a percentage of total body mass.

### Data Analysis

All data were analyzed using JMP 10.0.0 statistical software (SAS Institute Inc., 100 SAS Campus Drive, Cary, NC). We ensured that all residuals met parametric assumptions of a normal distribution using Shapiro-Wilk Goodness of Fit tests. The residuals from *Gryllus assimilis*' signalling parameters were mostly normally distributed (time spent signalling and interpulse duration were not). Half of *G. texensis*' signalling parameters were normally distributed for *G. texensis* (time spent signalling, chirp duration, interchirp duration, and pulses per chirp were not). We box-cox transformed the residuals from these six parameters to normalize them. Due to differences in *V*_max_ measurements between the date of assay, the residuals of the enzyme activities versus date of enzyme assay were taken and used for all further analyses with the aim of minimizing these differences.

To test our first hypothesis that intraspecific variation in sexual signalling is correlated with intraspecific variation in signalling muscle enzymes associated with carbohydrate and/or lipid metabolism, we ran a generalized linear model (GLM) for all 8 signalling parameters (16 models in total after accounting for both species). For each GLM we included as independent variables the activity of all six enzymes measured (PK, GP, CS, HOAD, TRE, and HK) and pronotum height. To test our second hypothesis that intraspecific variation in sexual signalling is correlated with intraspecific variation in capacity to accumulate and store carbohydrates and/or lipids, we ran a GLM for 8 signalling parameters (16 models in total after accounting for both species). For each GLM we included as independent variables the fuel store measures of carbohydrate, glycogen, and lipid contained in the thorax and abdomen. We did not include body size as an independent variable in these accumulation and storage models as all content measures were already corrected for body size. All GLMs were run as linear regressions with normal distributions and identity link functions.

## Results

### Gryllus assimilis

Males exhibited extensive variation in the amount of time they spent signalling. Some males never signalled acoustically during the 7 day recording period, while others averaged daily signalling times from just a few minutes to over 6 hours a day (Table 1). Males also varied in how they signalled (their fine scale signalling components), exhibiting 2- to 6-fold differences in most of their fine-scale signalling components (Table 1). Male body size was also highly variable (Table 1). Notably, males displayed a 2-fold difference in pronotum area and a 5-fold difference in body mass. Male muscle enzyme activity and energy stores also exhibited high intraspecific variation (Table 1). The most notable differences in enzyme activity were for GP and HOAD, with males displaying 22- and 16-fold differences, respectively. Males also displayed a 8-fold difference in TRE, a 5-fold difference in HK, and a 4-fold difference in CS activity. PK activity was the least variable. There were positive correlations between the activities of PK and GP, PK and HK, and HK and TRE (Table S1). Males exhibited 7-fold differences in their thoracic and abdominal glycogen stores. Male *G. assimilis* exhibited positive correlations between the abdominal energy stores of carbohydrates and glycogen (Table S1).

**Table 1:** Variation among male *G. assimilis* in their long distance acoustic mate attraction signals, enzyme activities, thoracic and abdominal percentages of carbohydrate, glycogen, and lipid, and morphology.

Trait	Mean	Median	Min	Max	St Error	CV	Difference	
TSC	94.00	73.55	0.00	364.06	8.51	86.8		
#Pulses	35052	24645	0	200207	3946.61	108.0		
Pulse Dur.	8.83	9.04	4.97	11.51	0.12	13.1	2.3	X
Inter-pulse Dur.	16.17	15.58	12.87	28.42	0.29	16.9	2.2	X
Chirp Dur.	104.79	105.38	49.67	180.74	2.04	18.4	3.6	X
Inter-chirp Dur.	1574.80	1573.04	575.86	2634.49	45.84	27.5	4.6	X
Pulses per Chirp	7.44	7.68	3.11	12.88	0.14	18.0	4.1	X
Carrier Freq.	4221.24	4215.95	3599.24	5324.89	43.91	9.8	1.5	X
Amplitude	41.12	43.33	11.32	63.99	1.27	29.0	5.7	X
PK activity	108.49	110.07	52.45	163.28	1.90	16.53	3.1	X
GP activity	1.42	1.18	0.23	5.02	0.10	70.41	22	X
CS activity	36.68	34.63	14.67	51.59	0.68	53.36	3.5	X
HOAD activity	68.25	71.26	7.98	131.13	3.18	44.74	16.4	X
TRE activity	3.30	3.04	1.06	8.36	0.15	42.17	7.9	X
HK activity	4.27	4.11	1.82	8.51	0.17	38.11	4.7	X
Thor. Carb (mg)	1.07	1.01	0.58	1.69	0.05	27.70	2.9	X
Thor. Gly (mg)	0.53	0.48	0.19	1.29	0.05	49.62	6.7	X
Thor. Lipid (mg)	14.68	13.81	5.73	30.28	1.00	37.48	5.3	X
Abdo. Carb (mg)	1.87	1.65	0.49	3.65	0.16	47.68	7.4	X
Abdo. Gly (mg)	2.05	1.56	0.64	5.85	0.23	61.23	9.1	X
Abdo. Lipid (mg)	9.38	8.87	3.33	19.07	0.85	49.76	5.7	X
Thor. Carb %	0.59	0.57	0.43	0.94	0.02	20.21	2.2	X
Thor. Gly %	0.29	0.25	0.11	0.74	0.02	45.32	6.5	X
Thor. Lipid %	20.38	19.26	8.36	39.87	1.31	35.31	4.8	X
Abdo. Carb %	1.29	1.17	0.49	2.62	0.10	43.67	5.3	X
Abdo. Gly %	1.43	1.23	0.54	3.54	0.15	56.71	6.6	X
Abdo. Lipid %	59.05	58.77	32.81	82.83	1.98	18.39	2.5	X
Body Mass (mg)	593.92	612.45	170.00	796.60	10.06	16.24	4.7	X
Muscle Mass (mg)	7.51	7.70	1.00	14.00	0.24	29.49	14.0	X
Pro. Area (mm^2^)	23.59	23.40	16.52	30.46	0.31	12.61	1.8	X
Pro. Length (mm)	3.98	3.98	3.31	4.74	0.03	8.34	1.4	X
Pro. Width (mm)	6.22	6.25	5.33	7.03	0.04	6.50	1.3	X
Head Width (mm)	5.17	5.16	4.52	5.98	0.03	5.59	1.3	X
Harp Area (mm^2^)	13.89	13.81	11.12	16.70	0.11	7.84	1.5	X
File Length (mm)	4.16	4.22	3.39	4.72	32.07	7.35	1.4	X

*Difference*  =  max/min values. Abbreviations: TSC (time spent signalling), Dur (duration), and Freq (Frequency), PK (pyruvate kinase), GP (glycogen phosphoylase), CS (citrate synthase), HOAD (β-hydroxyacyl-CoA dehydrogenase), TRE (trahalase), HK (hexokinase), Thor (thoracic), Abdo (abdominal), Pro (pronotum), Carb (carbohydrates), and Gly (glycogen). Enzyme activities are in U/g of muscle, where 1 U  =  1 µmol substrate/min. Units: TSC (min), Pulse Dur (msec), Inter-pulse Dur (msec), Chirp Dur (msec), and Inter-chirp Dur (msec), Carrier Freq (Hz), Amplitude (dB).

Males that signalled most often through the day were smaller (Table 2) but had higher thoracic glycogen stores (Table 3). This result was reversed in the abdomen, where males with lower abdominal glycogen stores signalled with higher effort. While variation in muscle enzyme activity did not correlate with variation in signalling effort, muscle enzyme variation was correlated with signal quality variation (Table 2). Specifically, males with higher GP activity signalled with shorter interpulse durations, at lower dominant frequencies, and produced significantly louder calls. Further, males with higher PK activity signalled produced signals with longer interchirp durations. Variation in the fine scale signalling components was not correlated with variation in muscle activity of CS, HOAD, and TRE, nor was it correlated with variation in fuel stores in either the thorax or abdomen (Table 3).

**Table 2:** Relationship between male *G. assimilis* acoustic mate attraction signalling parameters and enzyme activity assessed using generalized linear mixed models (df  =  7,91).

Behaviour	Parameter	Coefficient ± SE	X^2^	P
**TSC**	Whole Model		8.771	0.270
	PK	−0.23±0.51	0.192	0.661
	GP	4.08±9.98	0.167	0.683
	CS	−0.90±1.07	0.705	0.401
	HOAD	−0.44±0.29	2.322	0.128
	TRE	1.81±7.08	0.065	0.798
	HK	2.65±6.42	0.170	0.680
	Pronotum Height	−42.57±17.87	5.507	**0.019**
**Pulse Dur**	Whole Model		6.699	0.461
	PK	0.01±0.01	1.247	0.264
	GP	0.35±0.20	2.934	0.087
	CS	0.00±0.02	0.034	0.855
	HOAD	0.01±0.01	0.778	0.378
	TRE	−0.03±0.14	0.049	0.825
	HK	−0.12±0.13	0.800	0.371
	Pronotum Height	0.06±0.37	0.027	0.870
**Ipulse Dur**	Whole Model		13.104	0.070
	PK	0.00±0.02	0.022	0.882
	GP	−0.94±0.37	6.237	**0.013**
	CS	0.04±0.04	1.024	0.312
	HOAD	−0.02±0.01	3.799	0.051
	TRE	−0.28±0.26	1.156	0.282
	HK	0.21±0.24	0.787	0.375
	Pronotum Height	1.16±0.67	2.932	0.087
**Chirp Dur**	Whole Model		5.597	0.588
	PK	0.09±0.17	0.301	0.583
	GP	4.01±3.42	1.365	0.243
	CS	0.44±0.36	1.463	0.227
	HOAD	−0.13±0.10	1.632	0.202
	TRE	−0.40±2.41	0.028	0.868
	HK	−0.37±2.20	0.028	0.867
	Pronotum Height	0.16±6.19	0.001	0.980
**Ichirp Dur**	Whole Model		8.817	0.266
	PK	8.05±3.79	4.394	**0.036**
	GP	22.68±75.33	0.091	0.763
	CS	−10.25±8.02	1.619	0.203
	HOAD	2.76±2.16	1.616	0.204
	TRE	50.78±53.04	0.912	0.340
	HK	−42.63±48.55	0.767	0.381
	Pronotum Height	93.67±136.40	0.470	0.493
**PPChirp**	Whole Model		4.525	0.718
	PK	0.00±0.01	0.086	0.769
	GP	0.29±0.24	1.417	0.234
	CS	0.02±0.03	0.389	0.533
	HOAD	0.00±0.01	0.340	0.560
	TRE	0.12±0.17	0.471	0.493
	HK	−0.07±0.15	0.219	0.640
	Pronotum Height	−0.57±0.43	1.722	0.190
**Carr Freq**	Whole Model		9.098	0.246
	PK	−3.09±3.63	0.724	0.395
	GP	−167.76±72.04	5.267	**0.022**
	CS	−4.86±7.67	0.400	0.527
	HOAD	1.26±2.07	0.370	0.543
	TRE	−16.47±50.72	0.105	0.746
	HK	41.77±46.43	0.806	0.369
	Size PC1	127.61	0.952	0.329
**Amp**	Whole Model		10.496	0.162
	PK	0.04±0.10	0.137	0.711
	GP	5.75±2.06	7.470	**0.006**
	CS	0.12±0.22	0.319	0.572
	HOAD	−0.04±0.06	0.450	0.502
	TRE	−1.37±1.45	0.888	0.346
	HK	−0.14±1.33	0.011	0.918
	Pronotum Height	−3.24±3.73	0.749	0.387

**Table 3:** Relationship between *G. assimilis* acoustic mating signals and abdominal and thoracic content and assessed using generalized linear mixed models (df  =  6,91).

Behaviour	Parameter	Coefficient ± SE	X^2^	P
**TSC**	Whole Model		10.216	0.116
	Thor Carb	−134.38±142.64	0.888	0.346
	Thor Gly	218.62±96.78	5.103	**0.024**
	Thor Lipid	−21.66±12.67	2.921	0.087
	Ab Carb	208.28±201.71	1.066	0.302
	Ab Gly	−326.11±141.78	5.290	**0.021**
	Ab Lipid	33.14±18.45	3.225	0.073
**Pulse Dur**	Whole Model		7.944	0.242
	Thor Carb	−1.45±2.74	0.279	0.597
	Thor Gly	3.17±1.89	2.802	0.094
	Thor Lipid	−0.25±0.26	0.913	0.339
	Ab Carb	2.83±3.86	0.539	0.463
	Ab Gly	−5.20±2.76	3.550	0.060
	Ab Lipid	0.25±0.38	0.423	0.516
**Ipulse Dur**	Whole Model		6.313	0.389
	Thor Carb	−31.24±52.15	0.359	0.549
	Thor Gly	43.95±35.39	1.542	0.214
	Thor Lipid	−5.33±4.63	1.324	0.250
	Ab Carb	42.09±73.76	0.326	0.568
	Ab Gly	−67.68±51.84	1.704	0.192
	Ab Lipid	7.12±6.75	1.114	0.291
**Chirp Dur**	Whole Model		6.098	0.412
	Thor Carb	21.40±41.08	0.271	0.602
	Thor Gly	26.14±28.38	0.848	0.357
	Thor Lipid	−7.71±3.98	3.759	0.053
	Ab Carb	−23.26±57.90	0.161	0.688
	Ab Gly	−38.57±41.43	0.867	0.352
	Ab Lipid	10.41±5.69	3.356	0.067
**Ichirp Dur**	Whole Model		6.329	0.387
	Thor Carb	219.02±1006.71	0.047	0.828
	Thor Gly	126.51±695.59	0.033	0.856
	Thor Lipid	−8.30±97.43	0.007	0.932
	Ab Carb	−584.96±1419.01	0.170	0.680
	Ab Gly	88.86±1015.32	0.008	0.930
	Ab Lipid	−39.93±139.32	0.082	0.774
**PPChirp**	Whole Model		6.151	0.406
**Box Cox GT**	Thor Carb	1.60±3.04	0.276	0.599
	Thor Gly	1.31±2.10	0.390	0.532
	Thor Lipid	−0.52±0.29	3.165	0.075
	Ab Carb	−1.45±4.29	0.115	0.735
	Ab Gly	−2.06±3.07	0.450	0.502
	Ab Lipid	0.73±0.42	3.003	0.083
**Carr Freq**	Whole Model		3.855	0.696
	Thor Carb	−520.66±878.31	0.351	0.553
	Thor Gly	−232.41±606.87	0.147	0.702
	Thor Lipid	111.20±85.00	1.711	0.191
	Ab Carb	531.80±1238.03	0.185	0.668
	Ab Gly	402.48±885.82	0.206	0.650
	Ab Lipid	−145.94±121.55	1.441	0.230
**Amp**	Whole Model		3.087	0.798
	Thor Carb	−13.80±31.53	0.192	0.662
	Thor Gly	20.59±21.78	0.894	0.344
	Thor Lipid	0.28±3.05	0.008	0.927
	Ab Carb	21.43±44.44	0.232	0.630
	Ab Gly	−31.05±31.80	0.953	0.329
	Ab Lipid	−0.43±4.36	0.010	0.921

### Gryllus texensis

Male *G. texensis* exhibited extensive intraspecific variation in the amount of time they spent signalling through the course of a day. Some males never signalled while others averaged signalling times from just a few minutes to over 12 hours a day (Table 4). Males also varied in how they signalled; these differences were most noticeable with chirp and interchirp duration, where males exhibited 25- and 18-fold differences, respectively. Males exhibited 2- to 3-fold differences in most other fine-scale signalling components (Table 4). Males were also highly variable in their overall size (Table 4). Notably, males displayed 2-fold differences in their pronotum area and body mass. Male muscle enzyme activity and energy stores also exhibited high intraspecific variation (Table 5). The most notable differences in enzyme activity were for GP, CS, HOAD, TRE, and HK, where males exhibited 20-, 9-, 8-, 7-, and 5-fold differences, respectively. PK activity was the least variable. Several of the enzyme activity measures were positively correlated (e.g., GP and TRE, GP and HK, and HK and TRE), while several others were negatively correlated (e.g., PK and HK, CS and TRE, and HOAD and TRE; Table S1). The most notable intraspecific differences in energy stores were in thoracic and abdominal glycogen stores, where males displayed 14-and 13-fold differences, respectively. There were positive correlations between glycogen in the thorax and carbohydrates and glycogen contents in the abdomen (Table S1).

**Table 4:** Variation among male *G. texensis* in their long distance acoustic mate attraction signals, enzyme activities, thoracic and abdominal percentages of carbohydrate, glycogen, and lipid, and morphology.

Trait	Mean	Median	Min	Max	St Error	CV	Difference	
TSC	87.64	15.79	0.00	756.93	20.68	187.3		
#Pulses	264562	58225	0	2362084	60502.59	181.5		
Pulse Dur.	7.70	7.85	5.06	11.14	0.17	15.1	2.2	X
Inter-pulse Dur.	13.79	13.92	7.41	17.10	0.21	10.7	2.3	X
Chirp Dur.	613.15	529.44	78.78	1992.68	50.90	58.1	25.3	X
Inter-chirp Dur.	414.56	303.38	83.23	1453.24	43.20	72.9	17.5	X
Pulses per Chirp	50.58	39.65	8.61	220.20	5.12	70.8	25.6	X
Carrier Freq.	5242.11	5265.70	4141.26	5658.09	38.45	5.1	1.4	X
Amplitude	59.75	61.12	36.46	79.46	1.55	18.2	2.2	X
PK activity	104.73	107.49	47.13	131.35	2.26	15.87	2.8	X
GP activity	2.17	2.13	0.23	4.56	0.15	53.75	20	X
CS activity	38.59	41.16	5.56	51.38	1.04	19.76	9.2	X
HOAD activity	81.23	83.24	20.52	156.21	2.67	26.05	7.6	X
TRE activity	5.01	4.56	2.05	13.38	0.28	45.01	6.5	X
HK activity	8.64	8.21	3.80	20.52	0.41	37.46	5.4	X
Thor. Carb (mg)	1.01	0.83	0.35	3.02	0.10	54.85	8.5	X
Thor. Gly (mg)	1.24	0.82	0.29	4.59	0.20	90.84	15.6	X
Thor. Lipid (mg)	15.47	14.67	5.73	36.45	1.21	43.39	6.4	X
Abdo. Carb (mg)	1.76	1.58	0.57	4.47	0.16	51.15	7.8	X
Abdo. Gly (mg)	3.14	2.31	0.65	14.96	0.52	91.59	22.9	X
Abdo. Lipid (mg)	14.32	13.71	6.00	27.71	0.87	33.92	4.6	X
Thor. Carb %	0.60	0.52	0.29	1.39	0.05	42.29	4.7	X
Thor. Gly %	0.78	0.46	0.20	2.70	0.13	91.23	13.5	X
Thor. Lipid %	24.16	23.59	9.36	43.45	1.80	41.50	4.6	X
Abdo. Carb %	1.21	1.06	0.43	2.16	0.08	38.75	5	X
Abdo. Gly %	2.12	1.54	0.65	8.22	0.30	77.64	12.7	X
Abdo. Lipid %	38.20	36.09	21.49	68.33	1.98	28.83	3.2	X
Body Mass (mg)	540.89	530.30	361.80	823.30	13.76	20.19	2.3	X
Muscle Mass (mg)	6.18	5.85	1.30	11.80	0.20	25.17	9.1	X
Pro. Area (mm^2^)	22.86	22.80	14.17	33.20	0.47	16.28	2.3	X
Pro. Length (mm)	3.80	3.85	2.77	4.56	0.04	9.26	1.6	X
Pro. Width (mm)	6.06	6.06	4.97	7.31	0.06	8.12	1.5	X
Head Width (mm)	5.19	5.13	4.31	6.98	0.06	9.47	1.6	X
Harp Area (mm^2^)	10.64	10.60	8.32	13.79	0.15	10.56	1.7	X
File Length (mm)	3.57	3.55	3.06	4.38	42.36	9.20	1.4	X

The *Difference* column represents the fold difference between the minimum and the maximum values. Abbreviations are as follows (units of measurement at the end of parentheses): TSC (daily average time spent signalling, mins), #Pulses (daily average number of pulses), Pulse Dur (average pulse duration, ms), IP Dur (average interpulse duration, ms), Chirp Dur (average chirp duration, ms), IChirp Dur (average interchirp duration, ms), PPChirp (average number of pulses per chirp), Carr Freq (average dominant frequency of pulses, Hz), Amp (average amplitude of pulses, dB), PK (pyruvate kinase), GP (glycogen phosphoylase), CS (citrate synthase), HOAD (β-hydroxyacyl-CoA dehydrogenase), TRE (trahalase), HK (hexokinase), Thor (thoracic), Ab (abdominal), Pro (pronotum), Carb (carbohydrates), and Gly (glycogen). All enzyme activities are in U/g of muscle tissue, where 1 U  =  1 µmol substrate/min.

**Table 5:** Relationships between male G. texensis acoustic mate attraction signals and enzyme activity, assessed using generalized linear mixed models (df  =  7,62).

Behaviour	Parameter	Coefficient ± SE	X^2^	P
**TSC**	Whole Model		4.743	0.691
	PK	−0.01±0.18	0.001	0.972
	GP	−1.26±3.23	0.153	0.696
	CS	−0.22±0.40	0.307	0.580
	HOAD	0.33±0.16	4.001	**0.046**
	TRE	1.02±1.68	0.366	0.545
	HK	0.58±1.21	0.228	0.633
	Pronotum Height	−0.42±7.41	0.003	0.955
**Pulse Dur**	Whole Model		9.168	0.241
	PK	0.00±0.01	0.175	0.676
	GP	0.18±0.20	0.780	0.377
	CS	0.02±0.02	0.932	0.334
	HOAD	−0.01±0.01	2.047	0.153
	TRE	−0.24±0.11	5.057	**0.025**
	HK	0.06±0.07	0.665	0.415
	Pronotum Height	0.81±0.44	3.210	0.073
**Ipulse Dur**	Whole Model		3.301	0.856
	PK	0.02±0.01	2.333	0.127
	GP	−0.02±0.28	0.005	0.942
	CS	0.00±0.03	0.000	1.000
	HOAD	0.01±0.01	0.266	0.606
	TRE	−0.05±0.14	0.121	0.728
	HK	0.04±0.10	0.193	0.660
	Pronotum Height	−0.04±0.60	0.003	0.953
**Chirp Dur**	Whole Model		13.249	0.066
	PK	−2.27±2.81	0.649	0.421
	GP	4.88±52.60	0.009	0.926
	CS	0.36±5.89	0.004	0.951
	HOAD	−0.53±2.65	0.040	0.842
	TRE	28.83±27.18	1.112	0.292
	HK	10.93±18.66	0.342	0.559
	Pronotum Height	333.45±114.19	7.836	**0.005**
**Ichirp Dur**	Whole Model		12.842	0.076
	PK	4.30±1.61	6.670	**0.010**
	GP	−28.42±30.10	0.883	0.347
	CS	−1.86±3.37	0.302	0.583
	HOAD	3.24±1.52	4.341	0.037
	TRE	9.49±15.55	0.371	0.543
	HK	8.62±10.68	0.646	0.421
	Pronotum Height	−26.75±65.35	0.167	0.683
**PPChirp**	Whole Model		14.514	0.043
	PK	−0.22±0.23	0.904	0.342
	GP	−0.49±4.35	0.013	0.910
	CS	−0.01±0.49	0.000	0.987
	HOAD	−0.12±0.22	0.279	0.597
	TRE	3.00±2.25	1.751	0.186
	HK	1.05±1.54	0.460	0.498
	Pronotum Height	27.25±9.45	7.656	**0.006**
**Carr Freq**	Whole Model		17.334	**0.015**
	PK	−3.57±1.87	3.503	0.061
	GP	−50.96±35.10	2.062	0.151
	CS	−1.41±3.93	0.129	0.720
	HOAD	−1.72±1.77	0.938	0.333
	TRE	63.79±18.14	10.983	**0.001**
	HK	−17.92±12.45	2.027	0.155
	Size PC1	−57.81±76.19	0.572	0.449
**Amp**	Whole Model		11.468	0.120
	PK	−0.11±0.10	1.372	0.242
	GP	1.72±1.82	0.890	0.346
	CS	−0.07±0.20	0.120	0.729
	HOAD	−0.10±0.09	1.277	0.258
	TRE	0.35±0.94	0.141	0.707
	HK	−0.30±0.64	0.213	0.644
	Pronotum Height	8.76±3.94	4.699	**0.030**

Males that signalled with the highest effort had higher HOAD activity and higher thoracic carbohydrate stores compared to males that signalled less often (Tables 5 and 6). Further, males with higher thoracic carbohydrate stores also signalled with longer pulse durations and longer chirp durations. Males with higher abdominal carbohydrate and lipid stores signalled with shorter interpulse durations. Males with higher TRE activity signalled with shorter pulse durations but at higher carrier frequencies. Males with higher PK activity signalled with longer interchirp durations. Variation in other fine-scale signalling components was not correlated with variation in muscle activity of PK, GP, and HK or with variation in thoracic lipid stores or the glycogen stores in either the thorax or abdomen (Tables 5 and 6). Variation in fine-scale signalling components was, however, correlated with body size, as larger males signalled with more pulses per chirp, longer chirp durations, and louder.

**Table 6:** Relationships between male *G. texensis* acoustic mate attraction signals and abdominal and thoracic content, assessed using generalized linear mixed models (df  =  6,62).

Behaviour	Parameter	Coefficient ± SE	X^2^	P
**TSC**	Whole Model		11.549	0.073
	Thor Carb	38.56±15.89	5.891	**0.015**
	Thor Gly	5.78±10.28	0.316	0.574
	Thor Lipid	1.44±0.90	2.585	0.108
	Ab Carb	−4.81±9.43	0.261	0.610
	Ab Gly	1.47±4.13	0.127	0.721
	Ab Lipid	−0.44±0.95	0.212	0.645
**Pulse Dur**	Whole Model		4.626	0.593
	Thor Carb	447.30±151.75	8.689	**0.003**
	Thor Gly	−63.90±98.20	0.423	0.515
	Thor Lipid	15.84±8.57	3.414	0.065
	Ab Carb	−63.50±90.10	0.497	0.481
	Ab Gly	51.12±39.43	1.680	0.195
	Ab Lipid	0.23±9.10	0.001	0.980
**Ipulse Dur**	Whole Model		8.911	0.179
	Thor Carb	0.92±1.12	0.677	0.410
	Thor Gly	−1.24±0.74	2.821	0.093
	Thor Lipid	0.03±0.06	0.260	0.610
	Ab Carb	−1.94±0.93	4.350	**0.037**
	Ab Gly	0.12±0.28	0.182	0.670
	Ab Lipid	−0.28±0.10	8.528	**0.003**
**Chirp Dur**	Whole Model		7.252	0.298
	Thor Carb	837.26±421.00	3.955	**0.047**
	Thor Gly	127.47±272.43	0.219	0.640
	Thor Lipid	14.14±23.79	0.354	0.552
	Ab Carb	7.80±249.96	0.001	0.975
	Ab Gly	−27.72±109.40	0.064	0.800
	Ab Lipid	−22.15±25.24	0.770	0.380
**Ichirp Dur**	Whole Model		5.690	0.459
	Thor Carb	1704.73±1181.93	2.080	0.149
	Thor Gly	356.52±764.83	0.217	0.641
	Thor Lipid	28.47±66.78	0.182	0.670
	Ab Carb	−282.05±701.75	0.162	0.688
	Ab Gly	35.58±307.14	0.013	0.908
	Ab Lipid	−61.75±70.86	0.759	0.384
**PPChirp**	Whole Model		6.178	0.404
	Thor Carb	98.61±52.51	3.526	0.060
	Thor Gly	16.97±33.98	0.249	0.618
	Thor Lipid	1.36±2.97	0.211	0.646
	Ab Carb	−4.35±31.18	0.019	0.889
	Ab Gly	−2.82±13.65	0.043	0.836
	Ab Lipid	−1.97±3.15	0.390	0.532
**Carr Freq**	Whole Model		7.946	0.242
	Thor Carb	55.29±193.86	0.081	0.775
	Thor Gly	−199.64±126.96	2.473	0.116
	Thor Lipid	3.39±10.80	0.098	0.754
	Ab Carb	−63.77±160.35	0.158	0.691
	Ab Gly	43.54±47.79	0.830	0.362
	Ab Lipid	25.40±16.55	2.356	0.125
**Amp**	Whole Model		9.307	0.157
	Thor Carb	9.30±8.73	1.134	0.287
	Thor Gly	−0.80±5.72	0.020	0.889
	Thor Lipid	0.51±0.49	1.078	0.299
	Ab Carb	6.36±7.22	0.775	0.379
	Ab Gly	−1.65±2.15	0.587	0.443
	Ab Lipid	0.38±0.75	0.267	0.605

## Discussion

Crickets exhibited substantial intraspecific variation in their mate attraction signalling behaviour, in the enzyme activity of their signalling muscles, in their fuel stores, and in their body morphology. We found support for our hypothesis that an individual's ability to mobilize and/or metabolize fuels explains some of this signalling variation. Intraspecific signalling variation in chirping male *G. assimilis* was significantly linked to variation in body size and capacity to metabolise and store carbohydrates. Signalling effort was positively correlated with thoracic glycogen stores and negatively correlated with body size and abdominal glycogen stores. Further, *G. assimilis* males with increased glycogen phosphorylase activity in their signalling muscles produced signals with longer interpulse durations, at lower carrier frequencies, and at louder amplitudes, while males with increased pyruvate kinase activity produced signals with longer interchirp durations. Intraspecific signalling variation in trilling male *G. texensis* was also significantly linked to body size and capacity to metabolise and store lipids and carbohydrates. Signalling effort was positively correlated with HOAD activity in the signalling muscles and with thoracic free carbohydrate content. Further, *G. texensis* males with higher TRE activity and higher carbohydrate and abdominal lipid contents produced more attractive mating signals, signalling with longer pulse durations and shorter interpulse durations, but at higher dominant frequencies. Larger males produced longer chirps that contained more pulses and signalled at a higher amplitude. Overall, our findings suggest that the ability to mobilize and/or metabolize glycogen in *G. assimilis* and free carbohydrates and lipids in *G. texensis* may underlie some of the intraspecific signalling variation observed in nature.

Hill [9] called upon behavioural ecologists to determine whether expression of preferred traits reflects a capacity to remain near an optimal state. To do this, Hill [9] asked researchers to ascertain which cellular processes link preferred trait production to vital system functionality. Hill [9] hypothesized four alternative hypotheses: (1) the Resource Tradeoff Hypothesis where vital physiological pathways and preferred trait production both compete for the same resources; (2) the Mediator Hypothesis where a regulatory substance promotes preferred trait production while simultaneously depressing vital processes, or vice versa; (3) the Pathway Functionality Hypothesis where preferred trait production depends on the product of a vital physiological pathway; and (4) the Shared Pathway Hypothesis where both preferred trait production and vital processes share common pathways. Two of Hill's [9] four proposed hypotheses, the Pathway Function Hypothesis and the Shared Pathway Hypothesis, predict the positive correlations that we found between signalling and the capacity to store and use free carbohydrates and lipids in *G. texensis* and glycogen in *G. assimilis*. These two hypotheses both posit that instead of a trade-off between devoting energy to ornaments or to vital processes, the basic metabolic performance of a male is intrinsically linked to signalling whereby both processes either belong to the same metabolic pathway or use a shared metabolic pathway. Regardless of which of mechanism is operating, we hypothesize that signalling appears to be an honest indicator of condition without needing additional metabolic pathways over and above those of existing essential cellular processes.

### Alternative Mating Strategies

Our finding that metabolism of both carbohydrates and lipids is associated with signalling in *G. texensis* may help explain why some Texas field cricket males adopt a satellite behavioural strategy while others adopt a signalling strategy. Satellite males orient towards signalling males, sit nearby, and silently intercept and mate with attracted females [42]. While satellite males are capable of signalling, they rarely do so, instead exhibiting substantially lower average nightly signalling times than regular signallers [21], [23]. We compared enzyme activity in high- and low-effort signallers. *Gryllus texensis* males in the top quartile rank-ordered for daily time spent signalling (high-effort signallers) had significantly higher HOAD activities than males in the bottom rank-ordered quartile (non-signallers) (ANOVA: *F*_1,38_ = 4.598, *R^2^_adj_* = 0.089, *P* = 0.0388). The top and bottom quartiles did not, however, differ in their GP, TRE, or HK activities. These findings are commensurate with our hypothesis that high-effort signallers use a combination of lipids and carbohydrates, while low-effort signallers rely more on carbohydrates. The relationship is less clear with the remaining 50% of rank-ordered crickets that exhibit intermediate signalling effort.

### Signalling Muscle Metabolic Phenotypes

Based on the activity of enzymes measured and the ratios of enzymes involved in different pathways, both *G. texensis* and *G. assimilis* show high capacity to oxidise carbohydrate and lipid, a finding that is similar to the metabolic phenotypes of many insect muscles [43]. For example, the ratio of HOAD to CS mean activity (1.9 and 2.1 in *G. assimilis* and *G. texensis*) indicates that signalling muscle tissue is well poised for lipid oxidation. Ratios measured in insect flight muscle relying in part on lipid as oxidative fuel show ratios of 0.3 to 1.35 (locust [43]; crickets: [44]; soapberry bug: [45]; hawkmoth: [46]. The ratio of the glycolytic enzyme HK to CS mean activity (0.1 and 0.2 in *G. assimilis* and *G. texensis*) also suggests that signalling muscle tissue is capable of using both carbohydrate and lipid, or carbohydrate alone. In hawkmoths that power flight using either carbohydrate or lipid depending on dietary availability, the ratio is 0.07 [46]. In many species of bees that power flight using carbohydrate alone the ratio ranges from 0.03 to 0.24 [37], [47]. Signalling muscle therefore appears to be capable of fuelling oxidative metabolism using either carbohydrate or lipid. We reached the same conclusion in our study on *A. domesticus* (based on whole thorax measurements [38]).

It is important to note that even though the signalling muscle of *G. assimilis* and *A. domesticus* appear capable of utilizing lipid, their signalling behaviour seems to be primarily associated with carbohydrate use. We found significant correlations between signalling and PK activity in *A. domesticus* and GP activity in *G. assimilis,* suggesting that carbohydrate metabolism is recruited during signalling in both species. Thus, variation in signalling appears to be associated with variation in glycolytic pathway flux capacity, not variation in lipid metabolism in these two chirping species (*G. assimilis* and *A. domesticus*). These species may preferably power signalling using carbohydrate rather than lipid, because carbohydrate use is favored during short intense activity. Crickets that have sustained activity at moderate to high intensity, such as trilling *G. texensis*, seem to regularly process both lipid and carbohydrate.

It is also worth mentioning that protein availability during development might have profound implications on signalling muscle metabolic phenotypes. *Gryllus commodus* fed diets high in protein relative to carbohydrate throughout development and adulthood signal with greater effort than those fed diets with relatively higher levels of carbohydrate [28]. Nutrient availability during development is a vital aspect of an individual's condition and is necessary to grow to a large size (body size is fixed at adulthood). Since larger crickets signal with increase effort, diet during development could potentially be important in explaining the production of preferred traits and the maintenance of their variation. Nutrient availability during adulthood is also incredibly important. *Teleogryllus commodus* fed diets high in carbohydrates relative to protein signal with greater effort [48], while signalling effort is maximized by increasing total carbohydrate or protein intake regardless of dietary nutrient ratio in *G. veletis* [49]. These results reveal that the effects of nutrient availability on signalling and signalling muscle metabolic phenotypes may vary greatly between species.

Cellular metabolic phenotypes such as enzyme activities are labile traits. Nevertheless, interindividual variation in metabolic enzyme activity has been shown to relate to whole-animal performance traits. A recent example in vertebrates shows that interindividual variation in fish metabolic rate was associated with metabolic enzyme activity [50], and that active and resting metabolic rate measurements are repeatable in the same species [51]. Such association suggests that part of the interindividual variation in metabolic enzyme activity should be stable over time. In fact, muscle metabolic enzymes activities have been shown to be repeatable over repeated sampling in humans [52] and cattle [53], supporting the fact that measuring tissue metabolic phenotypes can characterize interindividual variability in energetic properties. In insects, recent work shows that interindividual variation in flight metabolic rate is repeatable over time [54], and such variation is associated with variation in flight muscle metabolic enzyme activities [54], [55]. It is noteworthy that aforementioned studies report interindividual variation in enzyme activity similar to the 2–3 fold range currently observed, supporting our conclusion that the range of variation we observed could impact whole-animal performance.

Signalling muscle metabolic phenotypes could be influenced by the flight capability of crickets. Field crickets often exhibit a hind wing dimorphism where micropterous (short hind wing) individuals have histolyzed flight muscles and are incapable of flight, while macropterous (long hind wing) individuals are capable of flight provided they have un-histolyzed flight muscles (macropterous flight muscles regularly undergo histolysis). Trade-offs between calling effort and investment in flight ability measured has been supported in crickets [56], while other studies suggests a positive relationship between these traits [57]. Regardless, these studies indicate the potential association between signalling and flight muscle properties.

Crickets with un-histolyzed flight muscles have higher overall metabolic activities [58] and higher enzyme activities in their flight muscles than crickets with histolyzed flight muscles (CS: 102, HOAD: 123 U g^−1^ in un-histolyzed muscle; CS: 17, HOAD: 19 U g^−1^ in histolyzed muscle; [44]. Our findings (CS: 37 and 39 U g^−1^ muscle; HOAD 68 and 81 U g^−1^ muscle in *G. assimilis* and *G. texensis* respectively) reveal that signalling muscle has greater aerobic capacity than histolyzed flight muscle, but lower values than flight capable morphs. We did not quantify the level of flight muscle histolysis in our study, but mesothoracic muscles do not seem to histolyze in adult male crickets [59]. Also, flight muscles tend to histolyze at a fairly young age in crickets: within 4 days of imaginal moult in *A. domesticus* [60], [61]; within 7 days of imaginal moult in *G. bimaculatus* [59]; and within 12 days of imaginal moult in *G. firmus* [58]. Our metabolic assays were conducted 14 days post imaginal moult, therefore all flight muscles should be in the same histolyzed state. Nevertheless, the extent to which flight muscle histolysis affects signalling muscle phenotypes and variation remains to be addressed.

### Conclusions

We comprehensively explored the physiological and biochemical traits correlated with signalling using multiple levels of organization (muscular enzymes and compartmentalized fuel stores). Intraspecific variation in time spent signalling and fine scale signalling parameters appears to be partially driven by variation in muscular enzyme activities and thoracic and abdominal fuel stores. Causational relationships between signalling and fuel need to be more fully explored. Combining studies that manipulate resource acquisition through diet with studies that manipulate resource allocation through imposing artificial selection on survival, signalling effort, etc. would provide insights into how stressed individuals differ from optimal in their biochemistry, life-history based allocation decisions, and preferred trait expression.

## Supporting Information

Table S1 **Matrix of Pearson correlations between enzyme activities for** ***G. assimilis*** **and** ***G. texensis.*** Each X-Y pair represents a single regression test. P-values are displayed above the diagonal line, Pearson correlation coefficients are displayed below the line. Significant p-values are bold, negative r values represent negative relationships. We compared six different enzymes and corrected for multiple tests using Benjamini and Yekutieli's false discovery rate (FDR_B-Y_) method; our FDR_B-Y_ corrected alpha was P<0.0125.
(DOCX)

## References

[1] Fisher RA (1930) The genetical theory of natural selection. Oxford: Clarendon Press.

[2] Houle D (1992) Comparing evolvability and variability of quantitative traits. Genetics 130: 195–204. 1732160 10.1093/genetics/130.1.195 PMC1204793

[3] Rowe L, Houle D (1996) The lek paradox and the capture of genetic variance by condition dependent traits. Proc R Soc B Biol Sci 263: 1415–1421.

[4] Tomkins JL, Radwan J, Kotiaho JS, Tregenza T (2004) Genic capture and resolving the lek paradox. Trends Ecol Evol 19: 323–328 10.1016/j.tree.2004.03.029 16701278

[5] Gould SJ (1975) Allometry in primates, with emphasis on scaling and the evolution of the brain. Contrib Primatol 5: 244–292. 803425

[6] Kotiaho JS, Marshall SD, Barrow JH, Jakob EM, Uetz GW (1999) Estimating fitness: comparison of body condition indices revisited. Oikos 87: 399–402.

[7] García-Berthou E (2001) On the misuse of residuals in ecology: testing regression residuals vs. the analysis of covariance. J Anim Ecol 70: 708–711 10.1046/j.1365-2656.2001.00524.x

[8] Tomkins JL, Simmons LW (2002) Measuring relative investment: a case study of testes investment in species with alternative male reproductive tactics. Anim Behav 63: 1009–1016.

[9] Hill GE (2011) Condition-dependent traits as signals of the functionality of vital cellular processes. Ecol Lett 14: 625–634 10.1111/j.1461-0248.2011.01622.x 21518211

[10] Alexander RD (1957) The taxonomy of the field crickets of the eastern United States (Orthoptera: Gryllidae: Acheta). Ann Entomol Soc Am 50: 585–602.

[11] Walker TJ (1957) Specificity in the response of female tree crickets (Orthoptera, Gryllidae, Oecanthinae) to calling songs of the males. Ann Entomol Soc Am 50: 626–636.

[12] Cade WH, Cade ESE (1992) Male mating success, calling and searching behaviour at high and low densities in the field cricket, *Gryllus integer* . Anim Behav 43: 49–56 10.1016/S0003-3472(05)80070-3

[13] Hedrick AV (1988) Female choice and the heritability of attractive male traits: an empirical study. Am Nat 132: 267–276.

[14] Roff D, Mousseau T, Howard D (1999) Variation in genetic architecture of calling song among populations of *Allonemobius socius*, *A. fasciatus*, and a hybrid population: drift or selection? Evolution 53: 216–224. 28565178 10.1111/j.1558-5646.1999.tb05347.x

[15] Cade WH (1981) Alternative male strategies: genetic differences in crickets. Science 212: 563–564. 17737211 10.1126/science.212.4494.563

[16] Bertram SM, Kemp DJ, Johnson JS, Orozco SX, Gorelick R (2007) Heritability of acoustic signalling time in the Texas field cricket, *Gryllus texensis* . Evol Ecol Res 9: 975–986.

[17] Walker TJ (1962) Factors responsible for intraspecific variation in the calling songs of crickets. Evolution 16: 407–428.

[18] Miyoshi AR, Zefa E, Martins LDP, Dias PGBS, Drehmer CJ, et al (2007) Stridulatory file and calling song of two populations of the tropical bush cricket *Eneoptera surinamensis* (Orthoptera, Gryllidae, Eneopterinae). Série Zool 97: 461–465.

[19] Alexander RD (1962) Evolutionary change in cricket acoustical communication. Evolution 16: 443–467.

[20] Martin SD, Gray DA, Cade WH (2000) Fine-scale temperature effects on cricket calling song. Can J Zool 78: 706–712 10.1139/cjz-78-5-706

[21] Cade WH (1991) Inter-and intraspecific variation in nightly calling duration in field crickets, *Gryllus integer* and *G. rubens* (Orthoptera: Gryllidae). J Insect Behav 4: 185–194.

[22] Bertram SM (2000) The influence of age and size on temporal mate signalling behaviour. Anim Behav 60: 333–339 10.1006/anbe.2000.1473 11007642

[23] Bertram SM, Warren PS (2005) Trade-offs in signalling components differ with signalling effort. Anim Behav 70: 477–484.

[24] Bertram SM, Fitzsimmons LP, McAuley EM, Rundle HD, Gorelick R (2011) Phenotypic covariance structure and its divergence for acoustic mate attraction signals among four cricket species. Ecol Evol 1: 1–15 10.1002/ece3.76 22408735 PMC3297187

[25] Cade WH (1979) The evolution of alternative male reproductive strategies in field crickets. In: Blum M, editor. Sexual selection and reproductive competition in insects. Academic Press, New York, New York.

[26] Crnokrak P, Roff DA (1995) Fitness differences associated with calling behaviour in the two wing morphs of male sand crickets, *Gryllus firmus* . Anim Behav 50: 1475–1481.

[27] Holzer B, Jacot A, Brinkhof MWG (2003) Condition-dependent signaling affects male sexual attractiveness in field crickets, *Gryllus campestris* . Behav Ecol 14: 353–359 10.1093/beheco/14.3.353

[28] Hunt J, Brooks RC, Jennions MD, Smith MJ, Bentsen CL, et al (2004) High-quality male field crickets invest heavily in sexual display but die young. Nature 432: 1024–1027 10.1038/nature03084 15616562

[29] Judge KA, Ting JJ, Gwynne DT (2008) Condition dependence of male life span and calling effort in a field cricket. Evolution 62: 868–878. 18194475 10.1111/j.1558-5646.2008.00318.x

[30] Simmons LW (1988) The calling song of the field cricket, *Gryllus bimaculatus* (De Geer): constraints on transmission and its role in intermale competition and female choice. Anim Behav 36: 380–394.

[31] Wagner WE (1996) Convergent song preferences between female field crickets and acoustically orienting parasitoid flies. Behav Ecol 7: 279–285 10.1093/beheco/7.3.279

[32] Popov A, Shuvalov V (1977) Phonotactic behavior of crickets. J Comp Physiol A 126: 111–126.

[33] Wagner WE, Murray A, Cade WH (1995) Phenotypic variation in the mating preferences of female field crickets, *Gryllus integer* . Anim Behav 49: 1269–1281 10.1006/anbe.1995.0159

[34] Prestwich KN (1994) The energetics of acoustic signaling in Anurans and Insects. Am Zool 34: 625–643 10.1093/icb/34.6.625

[35] Beenakkers AM, Van der Horst DJ, Van Marrewijk W (1984) Insect flight muscle metabolism. Insect Biochem 14: 243–260.

[36] Candy D, Becker A, Wegener G (1997) Coordination and integration of metabolism in inset flight. Comp Biochem Physiol 117: 497–512.

[37] Suarez RK, Darveau C-A, Welch KC, O'Brien DM, Roubik DW, et al (2005) Energy metabolism in orchid bee flight muscles: carbohydrate fuels all. J Exp Biol 208: 3573–3579 10.1242/jeb.01775 16155228

[38] Bertram SM, Thomson IR, Auguste B, Dawson JW, Darveau C-A (2011) Variation in cricket acoustic mate attraction signaling explained by body morphology and metabolic differences. Anim Behav 82: 1255–1261.

[39] Whattam EM, Bertram SM (2011) Effects of juvenile and adult condition on long-distance call components in the Jamaican field cricket, *Gryllus assimilis* . Anim Behav 81: 135–144.

[40] Lorenz MW (2003) Adipokinetic hormone inhibits the formation of energy stores and egg production in the cricket *Gryllus bimaculatus* . Comp Biochem Physiol Part B Biochem Mol Biol 136: 197–206 10.1016/S1096-4959(03)00227-6 14529746

[41] Kaufmann C, Brown M (2008) Determination of lipid, glycogen and sugars in mosquitoes. J Insect Physiol 54: 367–377. 18062987 10.1016/j.jinsphys.2007.10.007 PMC2267862

[42] Cade WH (1975) Acoustically orienting parasitoids: fly phonotaxis to cricket song. Science 190: 1312–1313.

[43] Beenakkers AM (1969) Carbohydrate and fat as a fuel for insect flight. A comparative study. J Insect Physiol 15: 353–361. 5779251 10.1016/0022-1910(69)90281-9

[44] Zera AJ, Sall J, Otto K (1999) Biochemical aspects of flight and flightlessness in Gryllus: flight fuels, enzyme activities and electrophoretic profiles of flight muscles from flight-capable and flightless morphs. J Insect Physiol 45: 275–285. 12770375 10.1016/s0022-1910(98)00123-1

[45] Winchell R, Dingle H, Moyes C (2000) Enzyme profiles in two wing polymorphic soapberry bug populations (Jadera haematoloma: Rhopalidae). J Insect Physiol 46: 1365–1373. 10878263 10.1016/s0022-1910(00)00055-x

[46] O'Brien DM, Suarez RK (2001) Fuel use in hawkmoth (*Amphion floridensis*) flight muscle: enzyme activities and flux rates. J Exp Zool 290: 108–114. 11471140 10.1002/jez.1040

[47] Skandalis DA, Roy C, Darveau C-A (2011) Behavioural, morphological, and metabolic maturation of newly emerged adult workers of the bumblebee, *Bombus impatiens* . J Insect Physiol 57: 704–711 10.1016/j.jinsphys.2011.02.001 21335010

[48] Maklakov AA, Simpson SJ, Zajitschek F, Hall MD, Dessmann J, et al (2008) Sex-specific fitness effects of nutrient intake on reproduction and lifespan. Curr Biol 18: 1062–1066. 18635354 10.1016/j.cub.2008.06.059

[49] Harrison S, Raubenheimer D, Simpson S, Godin J-G, Bertram S (2013) Towards a synthesis of frameworks in nutritional ecology: interacting effects of proteins, carbohydrates, and phsophorus on field cricket fitness. Ecol Lett: submitted. 10.1098/rspb.2014.0539 PMC4150310 25143029

[50] Norin T, Malte H (2012) Intraspecific variation in aerobic metabolic rate of fish: relations with organ size and enzyme activity in brown trout. Physiol Biochem Zool 85: 645–656. 23099462 10.1086/665982

[51] Norin T, Malte H (2011) Repeatability of standard metabolic rate, active metabolic rate and aerobic scope in young brown trout during a period of moderate food availability. J Exp Biol 214: 1668–1675. 21525312 10.1242/jeb.054205

[52] Simoneau JA, Lortie G, Boulay MR, Thibault MC, Bouchard C (1986) Repeatability of fibre type and enzyme activity measurements in human skeletal muscle. Clin Physiol 6: 347–356. 2943549 10.1111/j.1475-097x.1986.tb00240.x

[53] Harper G, Allingham P, Landsberg M (2000) Age and gender effects on two biochemical markers of muscle development in cattle. Asian-Australian J Anim Sci 13: 318–321.

[54] Darveau C-A, Billardon F, Bélanger K (2013) Intraspecific variation in flight metabolic rate in the bumblebee *Bombus impatiens*: repeatability and functional determinants in workers and drones. J Exp Biol: in press. 10.1242/jeb.091892 24198266

[55] Skandalis DA, Darveau C-A (2012) Morphological and physiological idiosyncrasies lead to interindividual variation in flight metabolic rate in worker bumblebees (*Bombus impatiens*). Physiol Biochem Zool 85: 657–670. 23099463 10.1086/665568

[56] Roff DA, Gelinas MB (2003) Phenotypic plasticity and the evolution of trade-offs: the quantitative genetics of resource allocation in the wind dimorphic cricket, *Gryllus firmus* . Evol Biol 16: 55–63. 10.1046/j.1420-9101.2003.00480.x 14635880

[57] Bertram SM, Schade J, Elser JJ (2006) Signalling and phosphorus: correlations between mate signalling effort and body elemental composition in crickets. Anim Behav 72: 899–907.

[58] Zera AJ, Sall J, Grudzinski K (1997) Flight-muscle polymorphism in the cricket *Gryllus firmus*: muscle characteristics and their influence on the evolution of flightlessness characteristics and their influence on the evolution of flightlessness. Physiol Zool 70: 519–529. 9279919 10.1086/515865

[59] Shiga S, Kogawauchi S (1991) Flight behaviour and selective degeneration of flight muscles in the adult cricket (*Gryllus bimaculatus*). J Exp Biol 155: 661–667.

[60] Chudakova I, Bocharova-Messner O (1968) Endocrine regulation of the condition of the wing musculature in the image of the house cricket (*Acheta domestica*). Dokl Akad Nauk SSSR 179: 489–492. 5745534

[61] Srihari T, Gutmann E, Novak V (1975) Effect of ecdysterone and juvenoid on the developmental involution of flight muscles in *Acheta domestica* . J Insect Physiol 21: 1–8. 1120906 10.1016/0022-1910(75)90062-1

[62] Benjamini Y, Yekutieli D (2001) The control of the false discovery rate in multiple testing under dependency. Ann Stat 29: 1165–1188.

