# Supplementary material for: Body Morphology, Energy Stores, and Muscle Enzyme Activity Explain Cricket Acoustic Mate Attraction Signaling Variation
Source: PLoS One. 2014 Mar 7;9(3):e90409. doi: 10.1371/journal.pone.0090409 (PMC3946518; doi:10.1371/journal.pone.0090409)
Supplement: Table S1 — Matrix of Pearson correlations between enzyme activities for G. assimilis and G. texensis. Each X-Y pair represents a single regression test. P-values are displayed above the diagonal line, Pearson correlation coefficients are displayed below the line. Significant p-values are bold, negative r values represent negative relationships. We compared six different enzymes and corrected for multiple tests using Benjamini and Yekutieli's false discovery rate (FDRB-Y) method; our FDRB-Y corrected alpha was P<0.0125. (DOCX) [file pone.0090409.s001.docx]

Table S1: Matrix of Pearson correlations between enzyme activities for *G. assimilis* and *G. texensis.* Each X-Y pair represents a single regression test. P-values are displayed above the diagonal line, Pearson correlation coefficients are displayed below the line. Significant p-values are bold, negative r values represent negative relationships. We compared six different enzymes and we corrected for multiple tests using Benjamini and Yekutieli’s [62] false discovery rate (FDR_B-Y_) method; our FDR_B-Y_ corrected alpha was P<0.0125.

|  |  | **PK** | **GP** | **CS** | **HOAD** | **TRE** | **HK** | **Thor Carb** | **Thor Gly** | **Thor Lipid** | **Ab Carb** | **Ab Gly** | **Ab Lipid** |
| --- | --- | --- | --- | --- | --- | --- | --- | --- | --- | --- | --- | --- | --- |
| ***G. assimilis*** | **PK** |  | **0.001** | 0.929 | 0.915 | 0.792 | **<0.001** | 0.601 | 0.714 | 0.661 | 0.152 | 0.045 | **<0.001** |
|  | **GP** | **0.349** |  | 0.203 | 0.932 | 0.122 | 0.033 | 0.460 | 0.148 | 0.308 | 0.032 | **0.001** | 0.613 |
|  | **CS** | 0.009 | 0.133 |  | 0.398 | 0.246 | 0.728 | 0.953 | 0.501 | 0.733 | 0.563 | 0.757 | 0.278 |
|  | **HOAD** | -0.011 | -0.009 | 0.089 |  | 0.798 | 0.660 | 0.814 | 0.953 | 0.232 | 0.509 | 0.641 | 0.725 |
|  | **TRE** | 0.028 | 0.162 | 0.121 | -0.027 |  | **<0.001** | 0.478 | 0.431 | 0.503 | 0.262 | 0.171 | 0.035 |
|  | **HK** | **0.383** | 0.221 | 0.037 | 0.046 | **0.543** |  | 0.386 | 0.831 | 0.731 | 0.502 | 0.275 | 0.004 |
|  | **Thor Carb** | 0.100 | -0.140 | 0.011 | 0.045 | 0.135 | 0.164 |  | 0.551 | 0.518 | 0.369 | 0.527 | 0.637 |
|  | **Thor Gly** | 0.070 | 0.271 | 0.128 | 0.011 | -0.149 | 0.041 | 0.113 |  | 0.100 | 0.405 | 0.435 | 0.634 |
|  | **Thor Lipid** | -0.084 | -0.193 | 0.065 | -0.225 | -0.127 | -0.065 | 0.123 | 0.306 |  | 0.127 | 0.064 | 0.306 |
|  | **Ab Carb** | 0.268 | 0.393 | 0.110 | 0.126 | 0.212 | 0.128 | 0.170 | 0.158 | -0.285 |  | **<0.001** | 0.244 |
|  | **Ab Gly** | 0.369 | **0.596** | 0.059 | 0.089 | 0.257 | 0.206 | 0.120 | 0.148 | -0.342 | **0.790** |  | 0.880 |
|  | **Ab Lipid** | **-0.618** | -0.096 | -0.205 | 0.067 | -0.387 | -0.511 | -0.090 | -0.090 | -0.193 | -0.219 | -0.029 |  |
| ***G. texensis*** | **PK** |  | **0.009** | 0.271 | 0.847 | 0.134 | **0.002** | 0.916 | 0.742 | 0.957 | 0.716 | 0.650 | 0.162 |
|  | **GP** | **-0.329** |  | 0.051 | 0.917 | **<0.001** | **<0.001** | 0.021 | 0.510 | 0.830 | 0.146 | 0.522 | 0.136 |
|  | **CS** | 0.142 | -0.249 |  | 0.373 | **0.001** | 0.392 | 0.915 | 0.613 | 0.592 | 0.882 | 0.954 | 0.130 |
|  | **HOAD** | 0.025 | -0.013 | 0.115 |  | **0.003** | 0.920 | 0.490 | 0.085 | 0.073 | 0.394 | 0.013 | 0.766 |
|  | **TRE** | -0.193 | **0.479** | **-0.412** | **-0.368** |  | **0.001** | **0.002** | 0.870 | 0.762 | 0.199 | 0.563 | 0.686 |
|  | **HK** | **-0.387** | **0.573** | -0.111 | 0.013 | **0.414** |  | 0.342 | 0.643 | 0.986 | 0.868 | 0.840 | 0.972 |
|  | **Thor Carb** | -0.020 | 0.413 | -0.020 | -0.129 | **0.541** | 0.177 |  | 0.510 | 0.400 | 0.362 | 0.068 | 0.741 |
|  | **Thor Gly** | 0.062 | 0.123 | -0.094 | 0.315 | -0.031 | 0.087 | -0.123 |  | 0.395 | 0.003 | **<0.001** | 0.766 |
|  | **Thor Lipid** | 0.010 | -0.040 | 0.100 | 0.326 | -0.057 | 0.003 | -0.157 | 0.158 |  | 0.210 | 0.358 | 0.110 |
|  | **Ab Carb** | 0.068 | 0.267 | 0.028 | -0.159 | 0.237 | 0.031 | 0.169 | -0.518 | -0.231 |  | 0.126 | 0.013 |
|  | **Ab Gly** | 0.085 | 0.120 | -0.011 | 0.442 | -0.108 | 0.038 | -0.332 | **0.805** | 0.171 | -0.281 |  | 0.867 |
|  | **Ab Lipid** | 0.257 | -0.274 | 0.278 | -0.056 | -0.076 | -0.007 | -0.062 | 0.056 | 0.293 | -0.440 | 0.031 |  |

Abbreviations are as follows: PK (pyruvate kinase), GP (glycogen phosphoylase), CS (citrate synthase), HOAD (β-hydroxyacyl-CoA dehydrogenase), TRE (trahalase), HK (hexokinase), Thor (thoracic), Ab (abdominal), Pro (pronotum), Carb (carbohydrates), and Gly (glycogen).
